# Antibodies against complement component C5 prevent antibody-mediated rejection after lung transplantation in murine orthotopic models with skin-graft-induced pre-sensitization

**DOI:** 10.1007/s11748-022-01844-0

**Published:** 2022-06-29

**Authors:** Yuki Shiina, Hidemi Suzuki, Atsushi Hata, Taisuke Kaiho, Hiroki Matsumoto, Takahide Toyoda, Yuichi Sakairi, Hironobu Wada, Shinichiro Motohashi, Ichiro Yoshino

**Affiliations:** 1 Department of General Thoracic Surgery, Chiba University Graduate School of Medicine, 1-8-1 Inohana, Chiba, Japan; 2 Department of Medical Immunology, Chiba University Graduate School of Medicine, Chiba, Japan; 3 Department of Thoracic Surgery, School of Medicine, International University of Health and Welfare, Narita, Japan

**Keywords:** Anti-complement component C5, Antibody-mediated rejection, Lung transplantation, Murine orthotopic model

## Abstract

**Objective:**

Antibody-mediated rejection (AMR) could induce acute or chronic graft failure during organ transplantation. Several reports have shown that anti-C5 antibodies are effective against AMR after kidney transplantation. However, few reports have assessed the efficacy of anti-C5 antibodies against AMR after lung transplantation. Therefore, this study aimed to evaluate the efficacy of this novel therapy against AMR after lung transplantation.

**Methods:**

BALB/c and C57BL/6 mice were used as donors and recipients. One group was pre-sensitized (PS) by skin transplantation 14 days before lung transplantation. The other group was non-sensitized (NS). Orthotopic left-lung transplantation was performed in both groups. Animals were killed at 2 or 7 days after lung transplantation and evaluated for histopathology, C4d immunostaining, and serum donor-specific antibodies (DSAs) (*n* = 5 per group). Isograft (IS) models with C57BL/6 mice were used as controls. To evaluate the efficacy of C5 inhibition, other animals, which received similar treatments to those in the PS group, were treated with anti-C5 antibodies, cyclosporine/methylprednisolone, anti-C5 antibodies/cyclosporine/methylprednisolone, or isotype-matched irrelevant control monoclonal antibodies (*n* = 5 per group).

**Results:**

Two days after lung transplantation, the NS group exhibited mild, localized graft-rejection features (rejection score: 0.45 ± 0.08, *p* = 0.107). The PS group exhibited AMR features with a significantly higher rejection score (2.29 ± 0.42, *p* = 0.001), C4d vascular-endothelium deposition, and substantial presence of serum DSA. On day 7 after lung transplantation, both groups showed extensive graft alveolar wall destruction, and high acute-rejection scores. Mice receiving anti-C5 antibodies or anti-C5/antibodies/cyclosporine/methylprednisolone demonstrated significantly lower acute-rejection scores (0.63 ± 0.23, *p* = 0.002; 0.59 ± 0.22, *p* = 0.001, respectively) than those receiving isotype control antibodies.

**Conclusions:**

Murine orthotopic allograft lung transplant models met the clinical diagnosis and pathogenesis classification criteria of AMR. In these models, anti-C5 antibodies suppressed AMR. Therefore, anti-C5 therapy may be effective against AMR after lung transplantation.

## Introduction

Antibody-mediated rejection (AMR) is a major concern among patients undergoing organ transplantation, leading to acute and chronic graft failure [1, 2], with a 50–70% mortality rate [3, 4]. The mechanisms of cellular rejection have been well elucidated. Several immunosuppressive agents, such as calcineurin inhibitors, are available. However, no effective treatments for AMR currently exist. Unlike AMR after kidney or heart transplantation [5, 6], AMR after lung transplantation is not well reported, possibly due to the absence of diagnostic tools. Finally, in 2016, the International Society for Heart and Lung Transplantation presented the first consensus report on AMR in lung transplantation, which defined its clinical diagnosis criteria, including the presence of circulating donor-specific antibodies (DSAs), positive C4d peritubular capillary staining and other histopathologic changes, as well as its pathogenetic classification [7].

Substantial serum DSA elevation is often observed during the early management of patients who have undergone lung transplantation, which has recently been shown to be associated with the incidence of chronic rejection [3]. However, how serum DSA is monitored and whether DSA-neutralizing treatments, such as anti-B cell agents or immunoglobulins, should be administered remains controversial. As such, elucidating the mechanisms of these phenomena is of high importance. A previous report demonstrated that rat allografts after lung transplantation had C4d deposition in their pulmonary capillaries [8]. C4d, a split product of complement component 4 (C4) that indicates an antigen–antibody reaction, is widely accepted as an AMR marker. In a previous study, we found that chronic rejection after lung transplantation is partially complement-dependent in a murine orthotopic lung transplant model [2].

In non-sensitized (NS) recipients, the production of de novo gamma-globulin (IgG) antibodies against transplanted organs through an allogeneic immune reaction takes approximately 14 days to occur. Therefore, the rejection observed in the acute phase is mostly caused by cellular responses. Kohei et al. [9] reported that in a murine renal transplantation model, alloantibodies and complements were activated immediately after transplantation. Serum DSA reached its peak within 2 weeks in the recipients pre-sensitized (PS) with skin allografts. Russell et al. [10] demonstrated that mice undergoing kidney transplantation after receiving skin graft exhibited poorer survival and earlier graft loss than those receiving kidney transplantation alone. Therefore, we hypothesized that pre-sensitization via skin graft also enhances AMR after lung transplantation.

The anti-C5 antibody binds to the C5 complement protein, blocking this terminal complement [11]. Therefore, anti-C5 therapy is considered effective against AMR. Previous reports have shown that eculizumab, a humanized monoclonal antibody, is effective against AMR after kidney transplantation, even in patients with DSA or ABO-blood-type incompatibility [12, 13]. Several studies, including a Phase 2, randomized, multicenter, two-arm clinical trial, reported that eculizumab reduced the treatment failure rate of kidney transplantation [14, 15]. A few reports have also shown that anti-C5 antibodies prevent acute vascular rejection and prolong allograft survival after heart transplantation in animal models [12, 16]. However, no clinical or experimental reports have examined the effectiveness of anti-C5 antibodies against AMR after lung transplantation. Therefore, this study aims to evaluate the effectiveness of anti-C5 antibodies in the prevention of AMR after lung transplantation in murine orthotopic models.

## Materials and methods

### Animals

BALB/c and C57BL/6 mice (25–30 g; Oriental Yeast, Japan) were used for skin and orthotopic left-lung transplantation. All animals were housed in the Biomedical Research Center, Chiba University, according to institutional guidelines. All the experiments were approved by the Chiba University of Medicine Institutional Care and Use Committee and were performed in accordance with standard guidelines as recommended by the Science Council of Japan (http://www.scj.go.jp/en/animal/index.html).

### Measurement of serum DSA

DSAs in recipient blood samples were detected by flow cytometric cross matches, as previously described [17]. Briefly, splenocytes obtained from donor spleens were prepared and exposed to recipient serum samples for 30 min, and subsequently exposed to fluorescent-labeled anti-mouse IgG (ab6785; Abcam, USA) or IgM (ab97229; Abcam, USA) antibodies, and finally analyzed with FACSVerse™ (Becton Dickinson, Franklin Lake, NJ, USA) [9]. Experimental values are expressed as the mean fluorescence intensity (MFI).

### Murine skin transplantations

Skin transplantations were performed as previously described [18] with some modifications. Briefly, the skin was harvested from the back of the donor mouse. The subcutaneous fat and connective tissue were removed. The skin was subsequently cut into 8-mm diameter circles, which were transplanted onto the recipient’s back with 5-0 nylon sutures. The animals were analyze 7, 14, 21, 28, or 70 days after skin transplantation (*n* = 5 per group). The control group consisted of untreated mice (*n* = 5).

### Murine orthotopic lung transplantations

Orthotopic transplantation of the left lung was performed as previously described [19]. Briefly, a left-lung graft was harvested from a donor mouse. Cuffs made using 20- to 26-gauge intravenous catheters were inserted into the vessels and the left bronchus of the graft. The recipient mouse subsequently underwent left pneumonectomy, and the graft was inserted. The PS group (*n* = 10) underwent lung transplantation 14 days after skin transplantation and was compared with the NS group (lung transplantation only, *n* = 10). The mice were harvested 2 or 7 days after lung transplantation (*n* = 5 per group) for histological examination of the lungs. No immunosuppressive, antibiotic, or anti-inflammatory therapy was administered at any time during the experimental period. As an experimental control for pathology, immunohistochemistry, cytokine expression, isograft (IS) lung transplantation with C57BL/6 mice was also performed (*n* = 5).

### *Removal of induced IgG with bacterial enzyme *(*Streptococcal immunoglobulin-degrading enzyme [IdeS]*)

To assess if skin-graft-induced IgG enhances AMR, the mice were administered intravenous IdeS, a streptococcal protease that cleaves IgG antibodies into F(ab’)2 and Fc fragments with a unique specificity [20]. Thirteen days after skin transplantation, the mice (*n* = 5) received IdeS (V8341, 500 U per mouse; Promega, WI, USA) and analyzed for serum DSA 1 day after IdeS treatment (14 days after skin transplantation). Thereafter, the mice, that received similar treatments as those in the PS group (*n* = 5), also received IdeS 1 day before lung transplantation, killed 2 days after lung transplantation, and evaluated for serum DSA, histology, and C4d staining of lung grafts.

### Anti-C5 treatment

To evaluate the impact of C5 inhibition, other lung and skin transplant recipients were treated with anti-mouse C5 monoclonal antibodies (anti-C5 mAb, BB5.1, 40 mg/kg/day; Hycult Biotech, PA, USA), cyclosporine (CyA, 10 mg/kg/day; TCI, Japan), and methylprednisolone (HYD, 1.6 mg/kg/day; TCI, Japan). The recipients (*n* = 5 per group) were treated with anti-C5, CyA/HYD, anti-C5/CyA/HYD or isotype-matched irrelevant control mAb (40 mg/kg/day; Wako, Japan). The treatments were intraperitoneally administered on days 1 and 2 after lung transplantation. All mice were harvested on day 2 after lung transplantation (16 days after skin transplantation).

### *C5a enzyme-linked immunosorbent assay *(*ELISA*)

Serum C5a was measured in mice treated with anti-C5 and CyA/HYD. C5a ELISA was performed using a mouse complement C5a ELISA kit, according to the manufacturer’s protocol (Abcam, Cambridge, England).

### Immunohistochemistry

Staining was performed on 4-µm tissue sections, as previously described [21]. Briefly, paraffin-embedded, formalin-fixed lung sections underwent antigen-retrieval treatment, followed by peroxide and protein blocks. Sections were incubated with the following primary antibodies: rabbit mAb (SP7) directed against mouse CD3 (ab16669; Abcam, USA), rabbit polyclonal antibodies directed against mouse CD46 (ab175397; Abcam, USA), and rabbit polyclonal antibodies directed against mouse C4d (HP8033; Hycult Biotech, USA). Subsequently, the sections were stained using the standard ultra-sensitive avidin–biotin complex peroxidase method. The sections were counterstained with hematoxylin. These samples were evaluated by four surgeons (HS, AH, YS, and TK) who were blinded to individual groups, and scored on a scale of 0–4 using the clinical classification criteria of lung-transplant rejection [22]. In these samples, capillary C4d staining was also scored as previously described [23]: 0, 1+ (< 10% of capillaries), 2+ (10–50%), or 3+ (> 50%).

### Cytometric bead array

Cell-free serum samples were assessed for cytokines using the CBA mouse Th1/Th2/Th17 Cytokine Kit (Becton Dickinson, Franklin Lakes, NJ, USA) and CBD mouse flex set IL-21 (Becton Dickinson, BD Biosciences Franklin Lakes, NJ, USA). The results were analyzed using FACSVerse and BD Cytometric Beads Array software version 1.3, according to the manufacturer’s instructions.

### Statistical analyses

Statistical analyses were performed using the JMP Pro software program (version 13; SAS Institute Inc., Tokyo, Japan). Data are expressed as the mean ± SEM. *p*-value < 0.05 was considered statistically significant.

## Results

### Macroscopic findings of skin grafts and DSA after skin transplantation

**Fig. 1:**
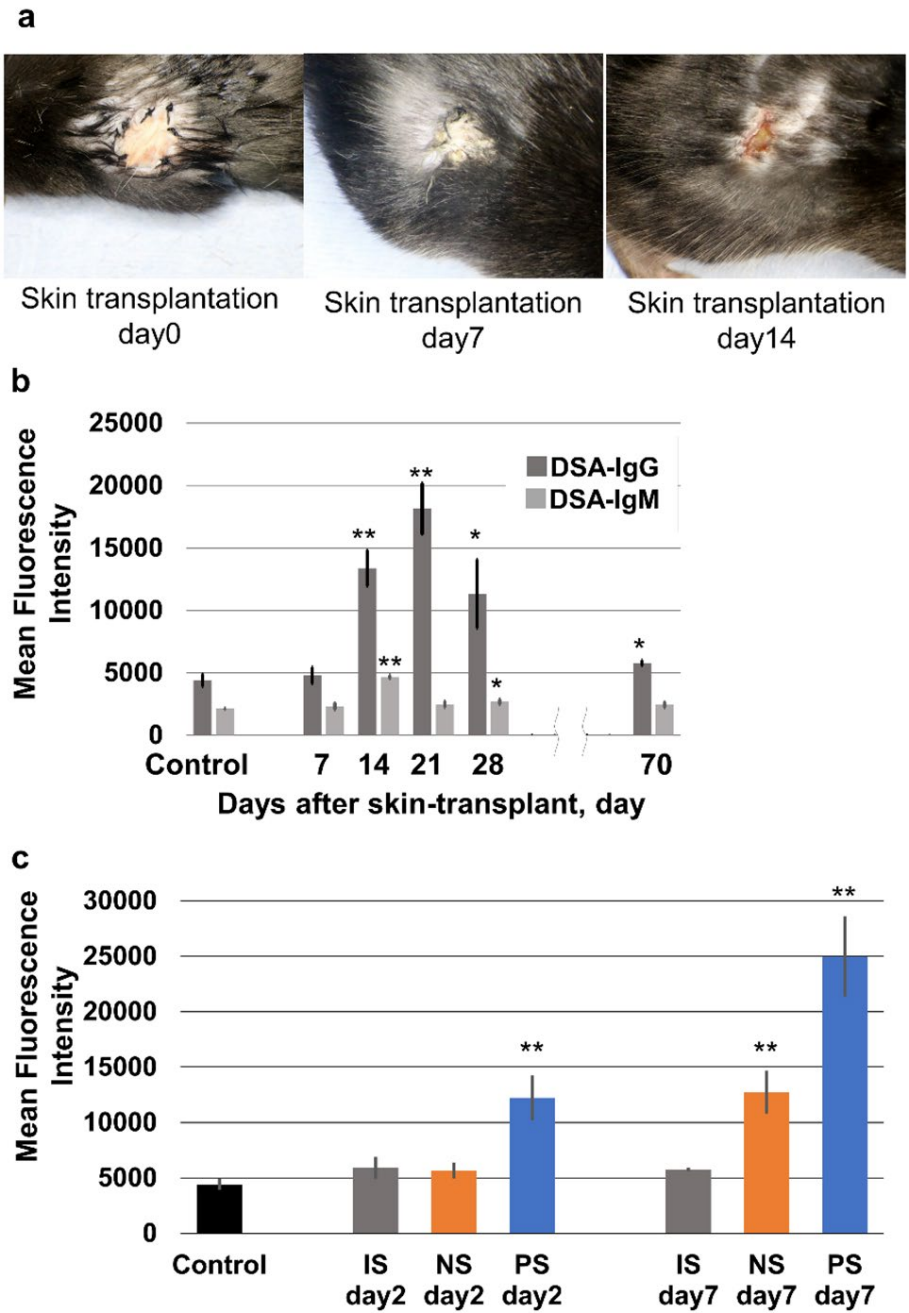
**a** Macroscopic findings of skin grafts and donor-specific antibody (DSA) levels after skin transplantation. Skin grafts were rejected 2 weeks after skin transplantation. **b** Serum DSA-IgG and DSA-IgM were observed on day 14 after skin transplantation. The control group consisted of mice without any treatments. **c** Serum DSAs were observed on day 7 after lung transplantation in the NS group, and on days 2 and 7 in the PS group. The NS group did not show increased serum DSA level on day 2. The control group consisted of mice without any treatments. **p <  0.01, comparison with the control group. *IS* isograft; *NS *non-sensitized; *PS* pre-sensitize

As skin graft rejection was defined as > 80% necrosis of the skin graft, all skin grafts were rejected 2 weeks after skin transplantation (Fig. 1a). DSA-IgG levels were significantly elevated 14 days after transplantation (13,369.0 ± 1442.7, *p* < 0.001), and peaked 21 days after transplantation (18,126.4 ± 2049.7, *p* < 0.001) (Fig. 1b). In contrast, DSA-IgM levels peaked 14 days after transplantation (4662.8 ± 148.6, *p* < 0.001). Based on this result, murine orthotopic lung transplantation in the PS group was performed 14 days after skin transplantation, when DSA-IgG levels were significantly elevated, in subsequent experiments.

### Macroscopic and pathological findings of acute rejection with acute-rejection scores of lung grafts

**Fig. 2:**
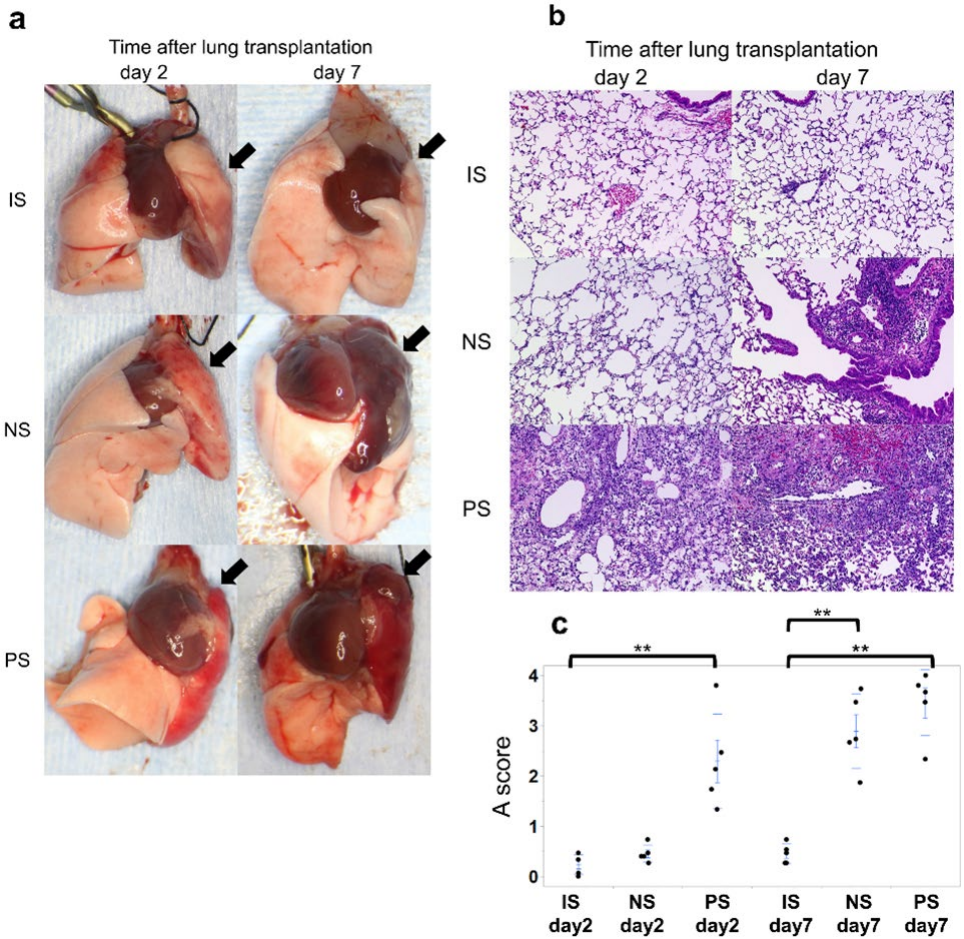
Macroscopic and pathological features and acute-rejection A score after lung transplantation in each group. **a** On day 2, the PS group macroscopically revealed wider congestion and atelectasis in their grafts (left lung) than the NS and IS groups. On day 7, the grafts exhibited wide congestion and atelectasis in both the PS and NS groups. **b** The PS group showed findings indicative of defuse rejection on days 2 and 7. However, the NS group showed findings indicative of mild rejection on day 2 and diffuse rejection on day 7. **c** Rejection A scores were significantly elevated on day 2 in the PS group. **p < 0.01, comparison with the control group. *IS* isograft; *NS* non-sensitized; *PS* pre-sensitized

On day 2 after lung transplantation, the PS group exhibited wider congestion and more atelectasis in their grafts compared to the NS group (Fig. 2a). In the histological findings on day 2, grafts from the NS group possessed only localized mononuclear cells surrounding small vessels and exhibited no other morphologic features of rejection, whereas those in the PS group exhibited dense perivascular mononuclear cells and alveolar damage (Fig. 2b). On day 7, the grafts in both groups had wide and diffuse leukocytic infiltration and extensive destruction of the alveolar walls.

Regarding acute-rejection scores, compared to control animals, the NS group demonstrated a significant increase in rejection scores on day 7 (2.89 ± 0.33, *p* < 0.001; Fig. 2c) but not on day 2, while the PS group showed a significant score increase, even on day 2 (2.29 ± 0.42, *p* = 0.001).

### C4d deposits by graft immunostaining

**Fig. 3:**
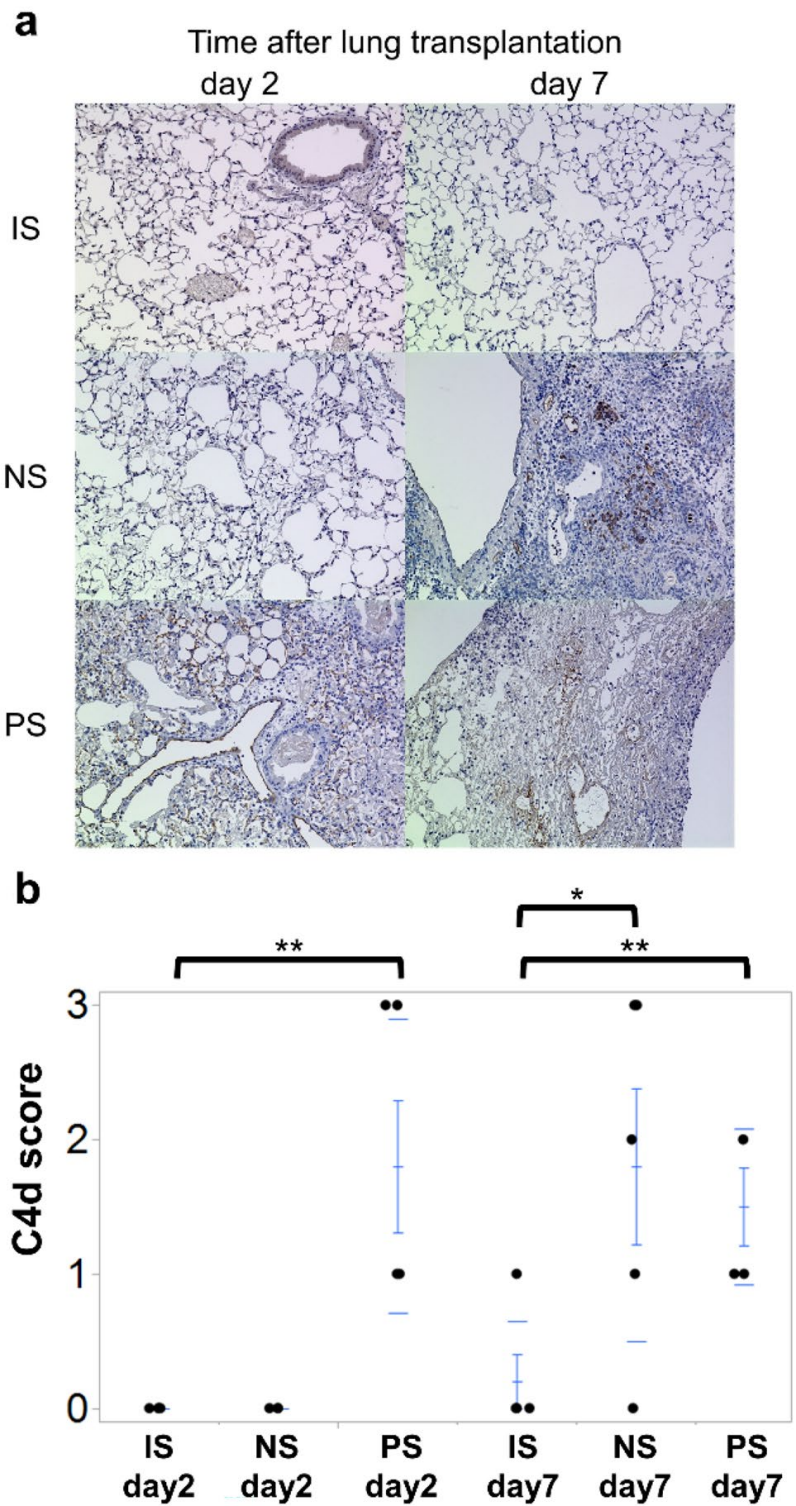
C4d staining of lung grafts in each group. **a** In the PS group, C4d deposits were observed in graft capillaries from an early stage (day 2). **b** The PS group demonstrated significantly high C4d scores on days 2 and 7. Nevertheless, the NS group showed significantly high C4d scores on day 7 only. **p < 0.01 comparison with the control group. *NS* non-sensitized; *PS* pre-sensitized

In the NS group, C4d deposits on the grafts were detected in the capillaries on day 7 but not on day 2 (Fig. 3a). In the PS group, C4d deposits were observed on day 2. Compared to control animals, C4d scores significantly increased on day 7 in the NS group (2.25 ± 0.48, *p* = 0.004), and on days 2 and 7 in the PS group (day 2: 1.80 ± 0.49, *p* = 0.008; day 7: 1.75 ± 0.48, *p* = 0.007; Fig. 3b).

### DSA analysis of after lung transplantation

DSA analyses after lung transplantation were performed for each group (Fig. 1c). In the NS group, serum DSA-IgG levels were normal on day 2 with an MFI of 5669.2 ± 729.0. However, they significantly increased on day 7 compared with those in the IS group (12,730.9 ± 256.38, *p* = 0.007). In contrast, the PS group exhibited a significant increase in serum DSA-IgG on both days 2 and 7 (day 2: 12,221.2 ± 2028.9, *p* = 0.023; day 7: 24,997.8 ± 3617.8, *p* < 0.001).

### Plasma cytokine analysis

**Fig. 4:**
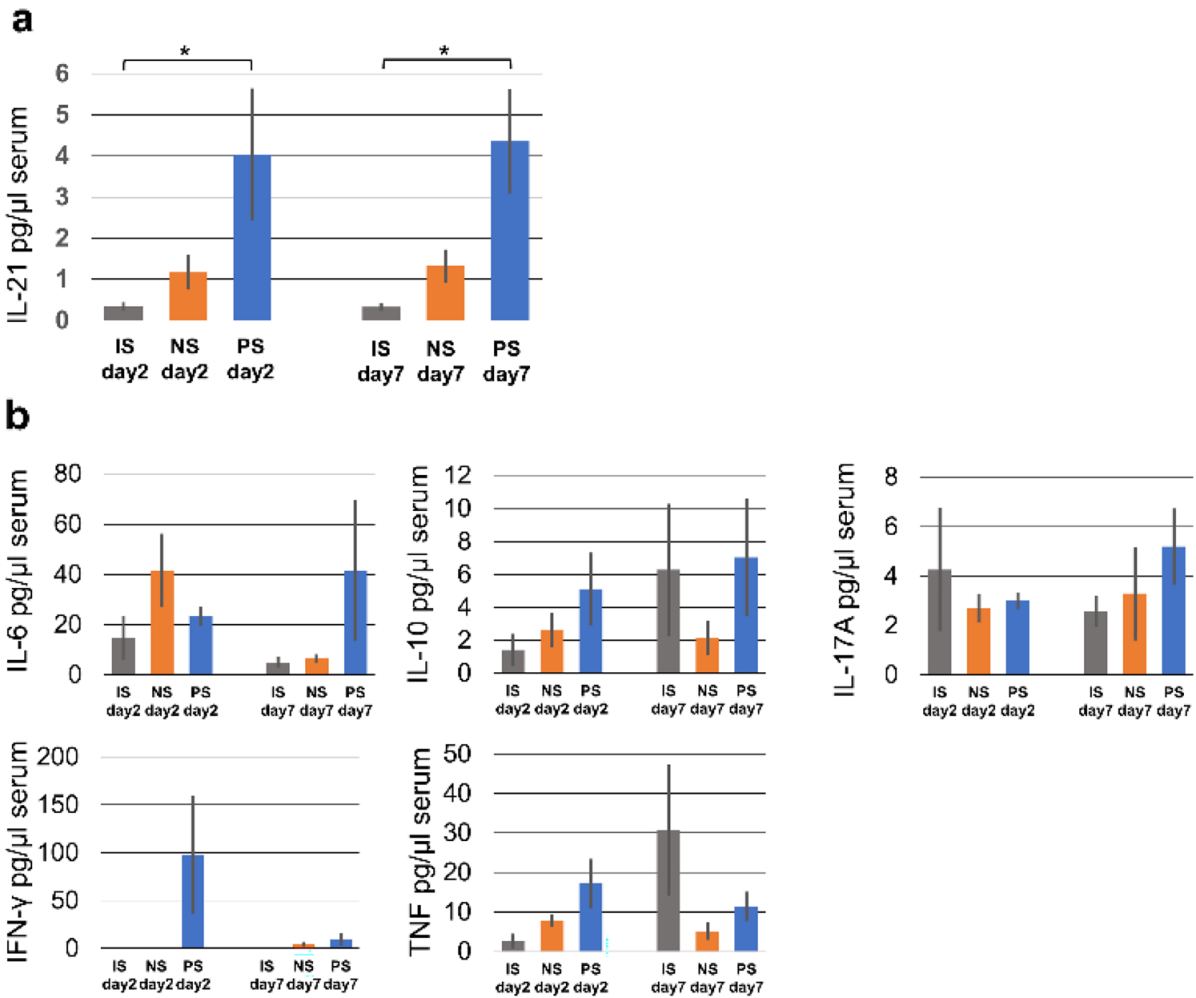
Serum cytokine production in each group. **a** Serum IL-21 was significantly higher in the PS group than in the NS group, indicating B cell activation. **b** No other cytokines exhibited significant differences among the groups. * and ** *p* < 0.05 and *p* < 0.01, respectively, comparison with the control group. *NS* non-sensitized; *PS* pre-sensitized

On both days 2 and 7 in the PS group, serum interleukin (IL)-21 level, a marker of B cell activation, was significantly higher than that in the IS group (day 2: 4.04 ± 1.05 vs. 0.34 ± 0.94, *p* = 0.036; day 7: 4.36 ± 0.90 vs. 0.29 ± 0.90, *p* = 0.013; Fig. 4a). In addition, there were no significant differences among the groups regarding other cytokines, such as IL-2, IL-4, IL-6, interferon (IFN)-*γ*, tumor necrosis factor (TNF), IL-17A, and IL-10 (Fig. 4b).

### DSA analysis and pathological findings in mice with IdeS

**Fig. 5:**
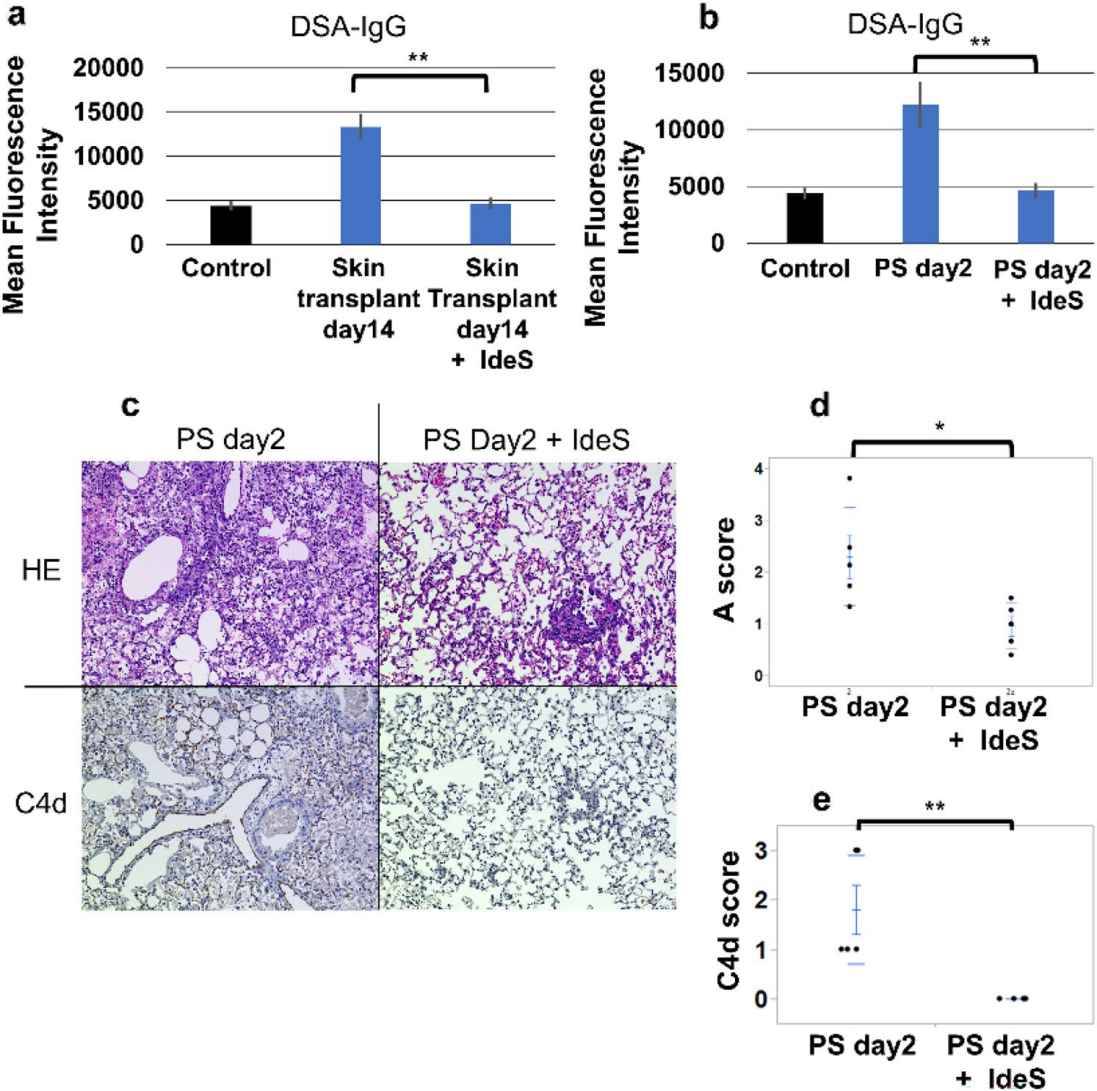
Donor-specific antibody (DSA) levels, pathological features, and rejection A score in IdeS-treated mice. **a** IdeS significantly reduced serum DSA-IgG in the mice 14 days after skin transplantation. **b** IdeS significantly decreased serum DSA-IgG in the mice 14 days after lung transplantation. **c** The mice with IdeS showed limited mononuclear cells around graft vessels and did not show C4d deposits on grafts. **d** The mice with IdeS showed significantly lower rejection A scores than those without IdeS. **e** The mice with IdeS exhibited significantly lower C4d scores than those without IdeS. The control group consisted of mice without any treatments. * and ** *p* < 0.05 and *p* < 0.01, respectively, comparison with the control group. *PS* pre-sensitized

Compared to mice without IdeS treatment DSA-IgG levels were significantly reduced in IdeS-treated mice 14 days after skin transplantation (13,369.0 ± 1442.7 vs. 4671.8 ± 656.9, *p* < 0.001; Fig. 5a). In the PS group, IdeS treatment also significantly reduced DSA-IgG levels (12,221.2 ± 2028.9 vs. 4639.4 ± 679.3, *p* = 0.008; Fig. 5b). In the PS group, the mice receiving IdeS showed considerably limited accumulation of mononuclear cells around the vessels on the grafts (Fig. 5c) and significantly lower rejection A scores than those without IdeS (0.78 ± 0.20 vs. 2.29 ± 0.42, *p* = 0.021; Fig. 5d). According to C4d staining, IdeS reduced C4d deposits on grafts (0.00 ± 0.00 vs. 1.80 ± 0.35, *p* < 0.001; Fig. 5c, e).

### Complement component 5 in serum

**Fig. 6:**
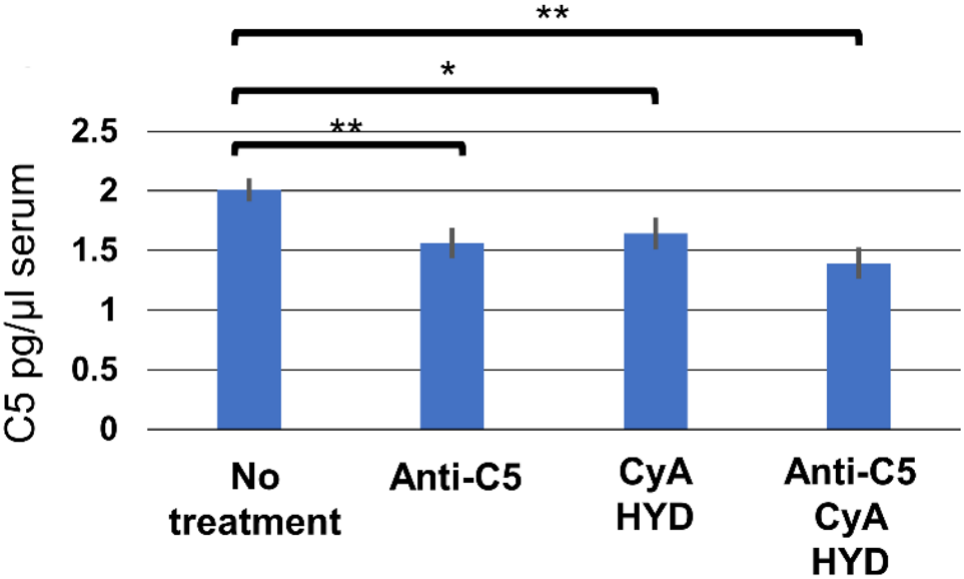
Serum C5 in each group 2 days after lung transplantation. Serum C5 levels were decreased in mice treated with anti-C5/CyA/HYD or anti-C5 alone compared with those without any treatments. * and ** *p* < 0.05 and *p* < 0.01, respectively, comparison with the control group. *CyA* Cyclosporin A; *HYD* hydrocortisone

The mice receiving anti-C5 treatment demonstrated significantly lower serum C5 levels than untreated counterparts (1472.50 ± 110.44 vs. 081.87 ± 75.17 pg/mL, *p* = 0.0022; Fig. 6). The mice with CyA/HYD and those with anti-C5/CyA/HYD also exhibited significantly lower C5 levels than untreated counterparts (CyA/HYD: 1683.91 ± 111.19 pg/mL, *p* = 0.018; anti-C5/CyA/HYD: 1297.59 ± 106.97 pg/mL, *p* < 0.001).

### Pathological findings of acute rejection and acute-rejection scores of lung grafts in the presence or absence of immune suppression

**Fig. 7:**
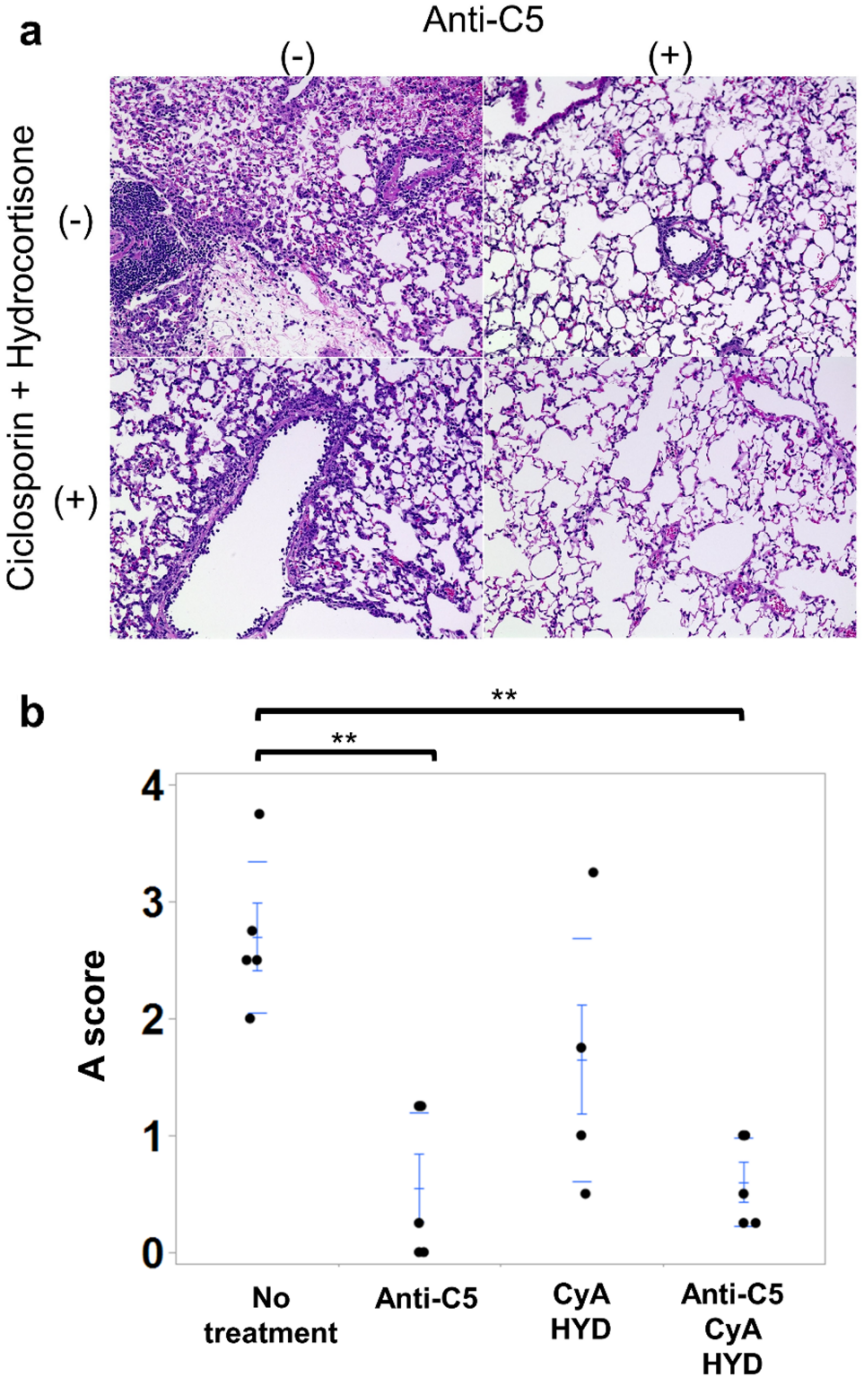
Macroscopic and pathological features and acute-rejection A scores after lung transplantation in the presence or absence of immune suppression. **a** The untreated group exhibited dense perivascular mononuclear cells, leukocytic infiltration, and alveolar damage on their lung grafts. The mice receiving anti-C5 or anti-C5/CyA/HYD showed mononuclear cells surrounding limited venules. **b** The mice receiving anti-C5 or anti-C5/CyA/HYD demonstrated significant decreases in rejection A scores (anti-C5: 0.63 ± 0.23, *p* = 0.002; anti-C5/CyA/HYD, 0.59 ± 0.22, *p* = 0.001) compared with untreated animals (2.58 ± 0.35). **p < 0.01, comparison with the control group. *CyA* Cyclosporin A; *HYD* hydrocortisone

The untreated group showed dense perivascular mononuclear cells, leukocytic infiltration, and alveolar damage to the graft lung (Fig. 7a). The animals receiving anti-C5 and those with anti-C5/CyA/HYD showed mononuclear cells surrounding a limited venule. Regarding acute-rejection A scores, both mice with anti-C5 only and anti-C5/CyA/HYD exhibited significant decreases in rejection A scores (anti-C5: 0.63 ± 0.23, *p* = 0.002; anti-C5/CyA/HYD: 0.59 ± 0.22, *p* = 0.001) compared with untreated counterparts (2.58 ± 0.35; Fig. 7b). Since the rejection A scores of the mice with CyA/HYD has a large variation (0.3–3.4), there was no significant difference between the mice with CyA/HYD and those in the other three groups.

### C4d deposits by graft immunostaining in the presence of absence of immune suppression

**Fig. 8:**
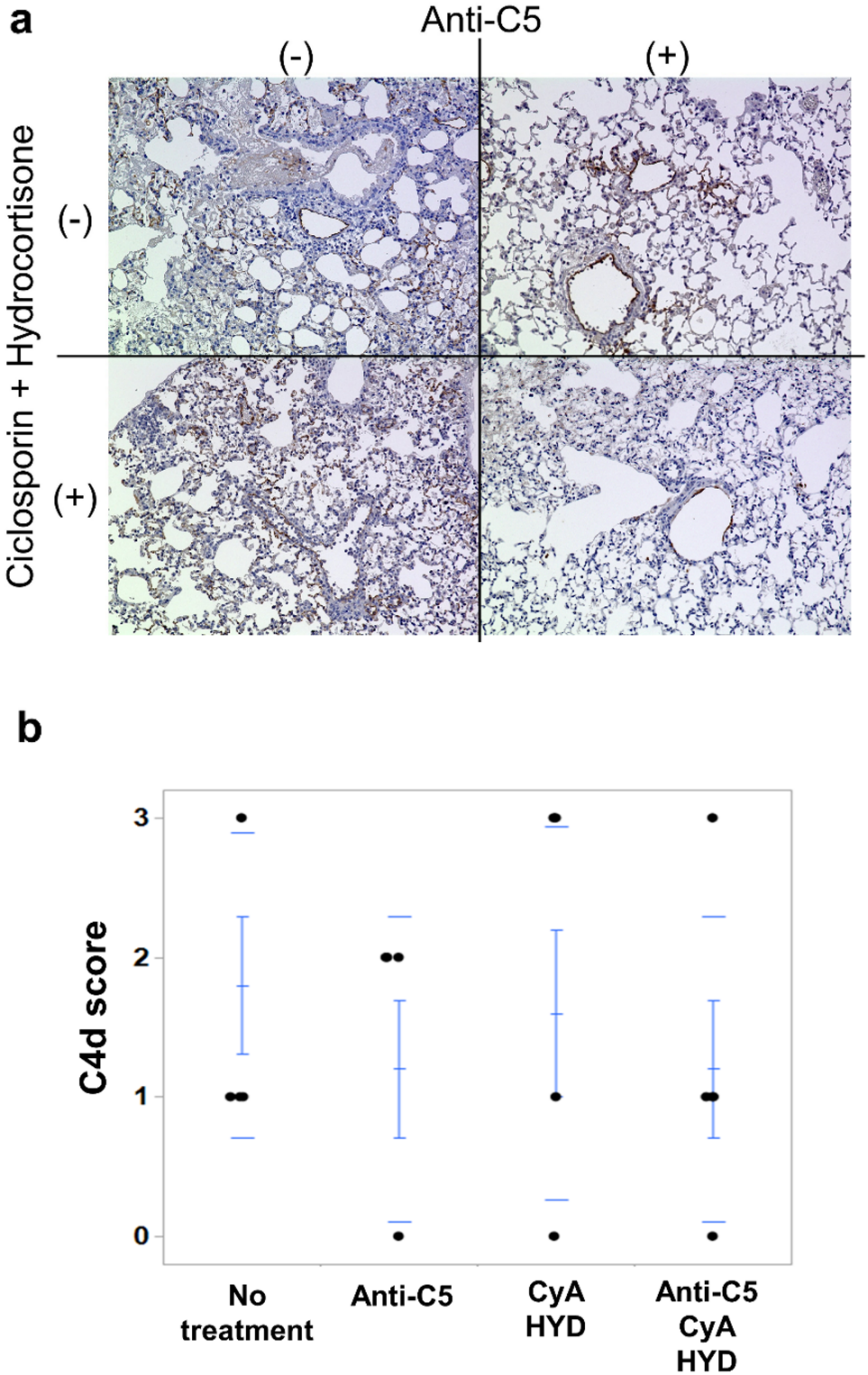
C4d staining of lung grafts with or without immune suppression. **a** C4d deposits on the grafts were detected in the capillaries in all groups. **b** There were no significant differences in C4d scores among all groups. *CyA* Cyclosporin A; *HYD* hydrocortisone

C4d deposits on the grafts were detected in the capillaries in all groups. Regarding the C4d score, there were no significant differences among the four groups. The C4d scores of the four groups varied from 0 to 3 (Fig. 8a, b).

### Plasma cytokine analysis

**Fig. 9:**
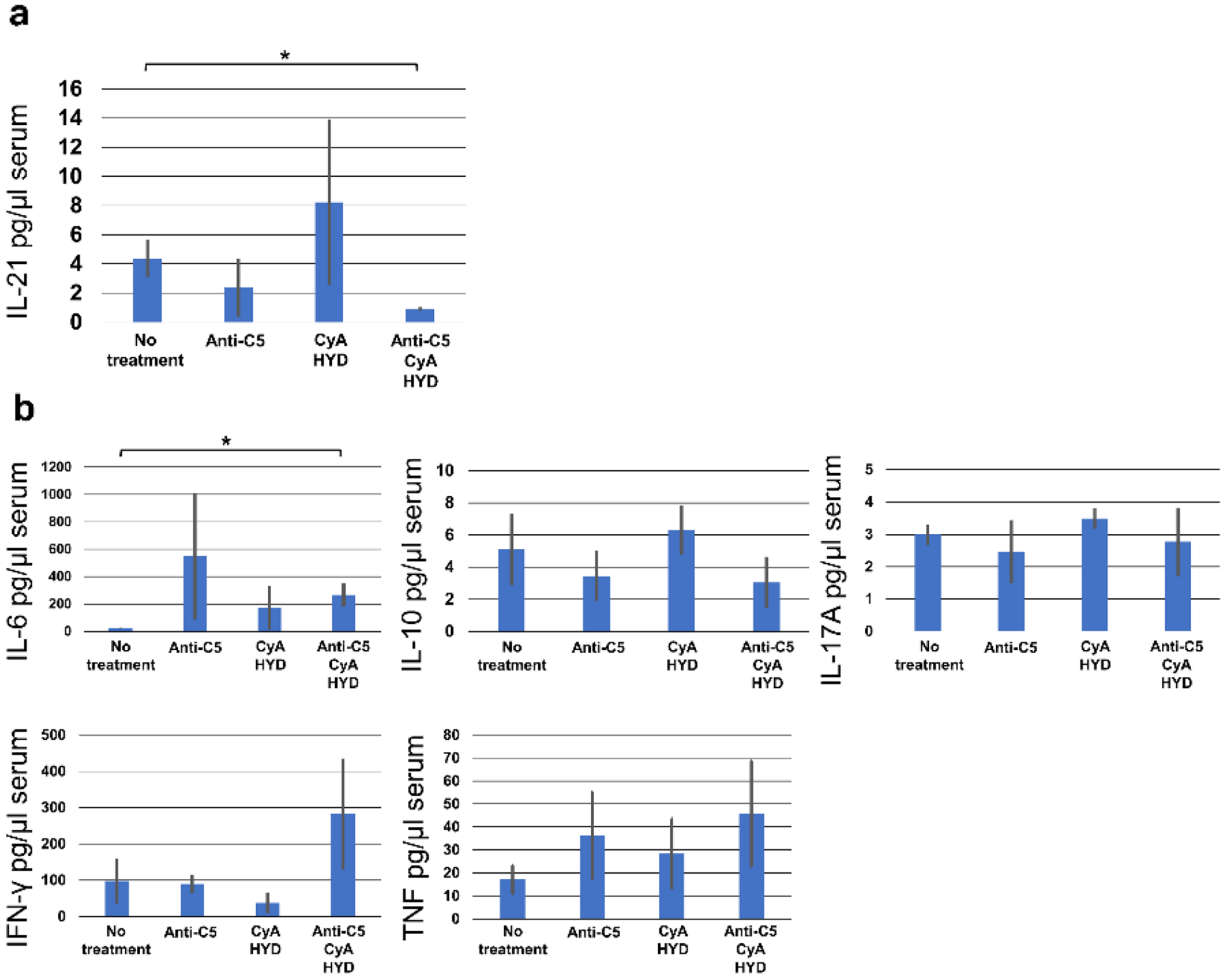
Serum cytokine production in each group. **a** Serum IL-21 was significantly lower in mice treated with anti-C5/CyA/HYD. **b** Serum IL-6 was significantly higher in mice treated with anti-C5/CyA/HYD. No other cytokine exhibited significant differences among the groups. *p < 0.05, comparison with the control group. *CyA* Cyclosporin A; *HYD* hydrocortisone

Serum IL-21 was significantly lower in the mice receiving anti-C5 and CyA/HYD (0.92 ± 0.12 pg/μL, *p* = 0.028) than in those in the no-treatment group (4.37 ± 1.31 pg/μL; Fig. 5a). There were no significant differences between the anti-C5 and untreated groups. Serum IL-6 was significantly higher in the mice with anti-C5 and CyA/HYD than in control counterparts (266.46 ± 83.67 vs. 23.23 ± 3.81 pg/μL, *p* = 0.026; Fig. 9b). Besides IL-21 and IL-6, there were no significant differences among the groups regarding IL-2, IL-4, IFN-γ, TNF, IL-17A, and IL-10 (Fig. 9b).

## Discussion

The murine orthotopic allograft lung transplant model met the AMR criteria. Pre-sensitization prior to lung transplantation potentially enhanced lung AMR. In addition, we demonstrated that anti-C5 therapy could be effective against AMR after lung transplantation in our murine models. To the best of our knowledge, this is the first report to reveal the efficacy of anti-C5 therapy for AMR after lung transplantation. Despite the use of immunosuppressive drugs, such as hydrocortisone and cyclosporin, graft rejection remains the main cause of mortality after lung transplantation [1, 2]. Patients with AMR after lung transplantation exhibited 25.0–28.6% and 25.0–42.9% mortality rates due to acute graft failure and chronic lung allograft disease (CLAD), respectively [4, 24]. Previous reports have revealed several AMR treatments or prevention strategies [12, 15, 25]. Berger et al. [25] reported that C1 inhibitor potentially improves post-transplantation outcomes. Lorant et al. [26] demonstrated that the kidney of a human leukocyte antigen-incompatible donor could be successfully transplanted after IdeS infusion. Bortezomib, a proteasome inhibitor that causes plasma cell apoptosis, was reported to be partially effective against AMR [27]. However, other reports have shown controversial outcomes of such treatments and prevention measures [28]. Hence, their effectiveness remains unclear. In addition, almost all reports mentioned treatment options for AMR after kidney transplantation, while few studies have investigated AMR after lung transplantation. In our study, anti-C5 treatment, but not by the combination of cyclosporine and methylprednisolone, suppressed AMR after lung transplantation in a murine model. Therefore, this study potentially contributes to the development of novel therapies for AMR after lung transplantation.

In addition, AMR diagnosis remains controversial in clinical lung transplantation. C4d deposition on renal grafts is an important AMR finding after renal transplantation. Many reports have shown an association between C4d deposition and poor graft survival after renal transplantation [29]. Nonetheless, the presence of C4d deposition on lung grafts of lung transplant patients with AMR remains controversial. Aguilar et al. [30] retrospectively reviewed 73 patients with definite or probable AMR and demonstrated that only 28 of 73 patients (38%) were C4d positive. Therefore, they concluded that C4d deposition is not necessary for AMR diagnosis in lung transplantation. Consistent with our study, PS mice, via pre-sensitization with skin graft, exhibited significantly higher C4d deposition scores with considerable disparities (1–3), despite their higher serum DSA levels and rejection scores. Unidentified complement-independent rejection factors might have contributed to AMR in lung transplantation.

One limitation of this study is that no respiratory functional evaluation, such as blood-gas analysis, was performed. However, macroscopic and pathological findings clearly indicated graft failure. In addition, in this study, lung transplantation was performed 14 days after skin transplantation because DSA appeared in the serum at this time. However, DSA was higher on day 21 after skin transplantation than on day 14. Therefore, the timing of lung transplantation remains disputable. Another limitation is that this study only evaluated AMR outcome during a limited period. Additional studies should be performed to evaluate the efficacy of anti-C5 therapy in preventing late-onset AMR or CLAD. Evaluation of the impact of C5 inhibition on animals at a more extended time course (7 days or more) after lung transplantation will be carried out. Notably, IL-6 is involved in chronic rejection after lung transplantation and showed significant increases in the mice receiving anti-C5 and CyA/HYD in our study. Therefore, analysis of the serum levels of IL-6 will also be conducted in future investigations.

## Conclusion

Anti-C5 therapy could be effective against AMR in murine orthotopic lung transplant models with skin-graft-induced pre-sensitization. This study potentially contributes to the development of novel therapies for AMR after lung transplantation. Further experimental research and clinical studies are required to assess the efficacy of anti-C5 therapy against AMR after lung transplantation.
